# Ten years of China’s new healthcare reform: a longitudinal study on changes in health resources

**DOI:** 10.1186/s12889-021-12248-9

**Published:** 2021-12-13

**Authors:** Jiang Chen, Zhuochen Lin, Li-an Li, Jing Li, Yuyao Wang, Yu Pan, Jie Yang, Chuncong Xu, Xiaojing Zeng, Xiaoxu Xie, Liangcheng Xiao

**Affiliations:** 1 Department of Medical Affairs, The First Affiliated Hospital, Sun Yat-sen University, Guangzhou, China; 2 School of Public Health, Fujian Medical University, Fuzhou, China

**Keywords:** Concentration index, Geo-Detector model, Geographic weighted regression, Moran’s I, Healthcare reform, Health resources

## Abstract

**Background:**

China launched a new round of healthcare-system reform in 2009 and proposed the goal of equal and guaranteed essential medical and health services for all by 2020. We aimed to investigate the changes in China’s health resources over the past ten years after the healthcare reform.

**Methods:**

Data were collected from the China Statistical Yearbook and China Health Statistics Yearbook from 2009 to 2018. Four categories and ten indicators of health resources were analyzed. A descriptive analysis was used to present the overall condition. The Health Resource Density Index was applied to showcase health-resource distribution in demographic and geographic dimensions. The global and local Moran’s I were used to assess the spatial autocorrelation of health resources. Concentration Index (CI) was used to quantify the equity of health-resource distribution. A Geo-Detector model and Geographic Weighted Regression (GWR) were applied to assess the association between gross domestic product (GDP) per capita and health resources.

**Results:**

Health resources have increased over the past ten years. The global and local Moran’s I suggested spatial aggregation in the distribution of health resources. Hospital beds were concentrated in wealthier areas, but this inequity decreased yearly (from CI=0.0587 in 2009 to CI=0.0021 in 2018). Primary medical and health institutions (PMHI) and their beds were concentrated in poorer areas (CI remained negative). Healthcare employees were concentrated in wealthier areas (CI remained positive). In 2017, the q-statistics indicated that the explanatory power of GDP per capita to beds, health personnel, and health expenditure was 40.7%, 50.3%, and 42.5%, respectively. The coefficients of GWR remained positive with statistical significance, indicating the positive association between GDP per capita and health resources.

**Conclusions:**

From 2009 to 2018, the total amount of health resources in China has increased substantially. Spatial aggregation existed in the health-resources distribution. Health resources tended to be concentrated in wealthier areas. When allocating health resources, the governments should take economic factors into account.

**Supplementary Information:**

The online version contains supplementary material available at 10.1186/s12889-021-12248-9.

## Background

According to the World Health Organization, equity is one of the most basic ethical principles of health-resource allocation [1]. Health-resource allocation reflects the distribution and flow of health resources in different regions. It is often used to measure the degree of health equity, which is regarded as an important part of social equity [2, 3]. Before the new healthcare reform, one of the most considerable problems the Chinese medical and healthcare system faced was the deficient and unbalanced distribution of health resources. The demographic and geographic maldistribution of healthcare resources is considered a prominent healthcare issue in China [4–6]. Studies have shown that inequality in the allocation of health resources is closely associated with increased disparities in health outcomes [7–9]. Frequently cited consequences of the unequal health-resource allocation are catastrophic health expenditures and impoverishment [10]. Focus on the unfair distribution of health resources is required to reduce health inequities [11]. In April 2009, China launched a new round of healthcare-system reform and set out to improve equitable access to medical services [12]. From 2009 to 2011, the government focused on increasing financial investment in the health sector to expand insurance coverage and build infrastructure. Since 2012, more emphasis has been placed on healthcare-delivery reform to increase health-service efficiency [10]. During this period, the government has issued a series of policies to promote equitable access to health resources, such as universal health insurance programs, zero-markup drug policy, patient-referral policy, medical alliance policy. In particular, the Healthy China 2030 program of the Chinese government that advocates to “accelerate the expansion of high-quality health resources and the balanced distribution of such resources among different regions” [13] has been regarded as a breakthrough for improving health [14].

Ten years after the new healthcare reform, the medical insurance system with almost universal coverage has been established [15], and the accessibility of medical services has been sharply improved [10]. Has a commensurate improvement in health-resource settings occurred? Has the allocation of health resources been gradually equal?

Previous studies have been conducted to assess health-resource allocation in China with different priorities. Such as emergency health resources [16, 17], primary medical and health institutions(PMHI) [18], comparison of the health resources and medical-service allocation between hospitals and PMHI [9], hospital beds [19], and health-workforce distribution [5]. The official description of health resources is usually divided into four categories, namely, healthcare institutions, beds, health personnel, and health expenditure [20]. In previous studies, they have often been cited as a proxy indicator of health resources, respectively [5, 9, 16–19]. To our knowledge, few scholars have conducted a systematic survey of China’s health resources within a span of ten years after the new healthcare reform so far. In particular, the involvement of health expenditure is scarce. In our study, we included the four categories of health resources. Through the research and analysis from the combination of these four aspects, we can have a more systematic and comprehensive understanding of China’s health resources. When assessing health-resource allocation, the results based on population density and geographic space showed a different situation [3, 9, 16, 17, 19]. Geographic inequalities in access to health resources have been addressed by previous studies as a challenging issue for many countries [4, 21–23]. The improvement of equity in spatial access to health resources is expected to potentially enhance the effectiveness of healthcare service utilization [4, 24, 25]. Thus, when describing the health-resource allocation, we compared it demographically and geographically, respectively. Measurements of health-resource distribution based on population density were more common, but it may ignore the effects of geography. The Health Resource Density Index (HRDI) was another tool that can be used. Compared with the assessment based on population density, it can mediate bias and influences owing to a single aspect of population or geographic area [9, 26]. Moran’s I was often applied to assess spatial autocorrelation. It included two types: Global Moran’s I and Local Moran’s I. The former was used to measure the general spatial autocorrelation and the spatial distribution of the research object, and the latter measured the local spatial autocorrelation and the cluster regions [27–29].

Many methods and indicators are used to study the equity of health-resource allocation, such as Concentration Index (CI), Lorenz Curve and Gini Coefficient, and Theil Index [30]. Each method and index have its own advantages and disadvantages, and the applicable conditions are not the same. Among these equity research methods, CI is extensively used to measure the degree of equity in the allocation of health resources associated with socioeconomic conditions [30, 31]. When measuring the equity of health-resource allocation, CI considers the social and economic conditions. It can accurately reflect the allocation of health resources to different social strata [32], which can help the government understand the allocation of health resources at all levels of society.

Gross domestic product (GDP) per capita is considered when calculating CI, and it has been addressed by previous studies as a factor affecting health-resource distribution [33, 34]. A Geo-Detector model, which is often used to measure similarities in the spatial distributions of two variables [35–37], has been developed to evaluate the spatial and temporal matching levels between GDP per capita and health resources at the provincial level. Geographic weighted regression (GWR) is a local regression based on geospatial weighting, which can effectively capture the impact of independent variables on outcomes in local areas [38]. It has been applied to assess the impact of GDP per capita on the distribution of health resources.

The present longitudinal study aimed to systematically investigate the changes in China’s health resources (including overall condition, spatiotemporal distribution, equity of allocation, and the association between GDP per capita and health resources) over the past ten years after the healthcare reform. Our findings are expected to comprehensively show the overview of changes in the distribution of health resources in China, and the results can be applied to provide implications on future spatial allocation of health resources and evidence-based health planning procedures.

## Methods

### Data sources

The indicators of health resources were adopted in accordance with the " Statistical Communique on the Development of China’s Health Undertakings " published by the China National Health Commission every year. These indicators are commonly cited in studies on health resources in China [5, 9, 16–19]. Hospitals and PMHI are the main places for diagnosis and treatment, and they account for the vast majority proportion of medical and healthcare institutions (more than 97% in 2018 [20]). Thus, they were included as indicators of healthcare institutions in the study. Correspondingly, their beds were also included. Health personnel refers to employees engaged in the healthcare institutions. In this study, licensed doctors (LD), registered nurses (RN), and healthcare employees (HCE) were included. Total health expenditure (THE) is usually divided into three types, including government health expenditure (GHE), social health expenditure (SHE), and Out-of-pocket payments (OOPs). Data for demographic, geographical, and socioeconomic including total population, geographical area, and GDP per capita were also included for the distribution assessment of health resources. These indicators are defined in the China Statistical Yearbook and China Health Statistical Yearbook and are listed in Additional file 1.

The data related to these indicators of 31 provinces, autonomous regions, and municipalities directly under the central government originate from China Statistical Yearbook and China Health Statistics Yearbook from the year 2009 to 2018. Due to inconsistent statistical standards, Hong Kong, Macau, and Taiwan were not included in this study.

### Study design

This study was divided into three steps.

### Description of the general situation

For the first step, we described the overall condition of health resources in China over the past ten years from four categories. Ten indicators including hospital, PMHI, hospital beds, PMHI beds, HCE, LD, RN, GHE, SHE, and OOPs were studied. In addition to the above ten indicators, we also introduced the calculation of Beds per 1,000 people, HCE per 1,000 people, GHE Per Capital, the proportion of OOPs in THE, and the proportion of THE in GDP for a overview of health resources.

### Demographic and geographic distribution of health resources

For the second step, we explored the demographic and geographic distribution of health resources. Since we introduced calculation methods of per capita or per thousand people in the comparison process, this was not applicable to healthcare institutions in practice. Therefore, in this process, we mainly compared three categories of indicators, namely beds, health personnel, and health expenditure. We used maps to visually present the distribution of beds (both in hospitals and PMHI per 1,000 people), HCE per 1,000 people, and THE per capita (refers to the ratio of THE in a year to the average population) between 2009 and 2018. We also included HRDI to present the integrated health-resource distribution from the aspects of population density and geographic area. HRDI was calculated as the geometric mean of health resources per 1,000 people and per square kilometers. The following formula was used to calculate HRDI [9, 26]:

$$HRDI=\sqrt{\left(Yi/\;Pi\right)\left(Yi/\;Ai\right)}$$

*Yi* represents the health resource of unit *I*; *Pi* represents the population of unit *I*, and *Ai* represents the area of unit *I*.

The results of HRDI were also presented by maps. To measure the global autocorrelation, we introduced the global Moran’s I index. The global Moran’s I is an important index to measure spatial autocorrelation with the range of -1 to 1 [39]. If Moran’s I is larger than 0, the resources had spatial disparity, indicating that a larger (smaller) resource corresponded with easier (harder) aggregation. If Moran’s I was smaller than 0, the resources had spatial heterogeneity, indicating that a larger (smaller) resource corresponded with less likelihood of aggregation. When Moran’ I was 0 (or P > 0.05), resources were randomly distributed in space and had no spatial correlation. When the global Moran’s I is statistically significant, the local Moran’s I can be further analyzed. The local Moran’s I can analyze whether an indicator in a local area has spatial correlation, which can be divided into two parts: (1) The level of an indicator in the region compared with the overall; (2) The level of indicators in the surrounding areas compared with the overall. The global Moran’s I and local Moran’s I can be calculated by:

$$Gobal Moran\text{'}s I=\frac{n}{{S}_{0}}\text{*}\frac{{\sum }_{i=1}^{n}{\sum }_{j=1}^{n}{\omega }_{ij}({y}_{i}-\overline{y})({y}_{j}-\overline{y})}{{\sum }_{i=1}^{n}{({y}_{i}-\overline{y})}^{2}}$$

$$Local Moran\text{'}s I={I}_{i}=\frac{({y}_{i}-\overline{y})}{{\sum }_{k=1}^{n}{\left({y}_{i}-\overline{y}\right)}^{2}/(n-1)}\text{*}\sum _{j\ne i}^{n}{w}_{ij}({y}_{j}-\overline{y})$$

Where *n* is the number of the province, *yi* and *yj* are the resource of province *i* and *j*, respectively, $\overline{y}$ is the mean of the resource of all provinces, ${S}_{0}={\sum }_{i=1}^{n}{\sum }_{j=1}^{n}{\omega }_{ij}$, and${\omega }_{ij}$ is the spatial weight value of province *i* and *j* calculated from the spatial distance using Euclidean distance. It should be noted that the data points used for the calculation of ${ \omega }_{ij}$ were different between global Moran’s I and local Moran’s I.

### Assessment of the equity and the association between economic factor and health resources

For the third step, we used CI to quantify the degree of inequality in the allocation of health resources. We introduced a Geo-Detector model and GWR to further measure the association between GDP per capita and health-resource distribution.

CI is recognized as a superior tool to measure the equality of health-resource allocation associated with socioeconomic status [32]. The following figure was used to demonstrate the definition of CI.

**Fig. 1:**
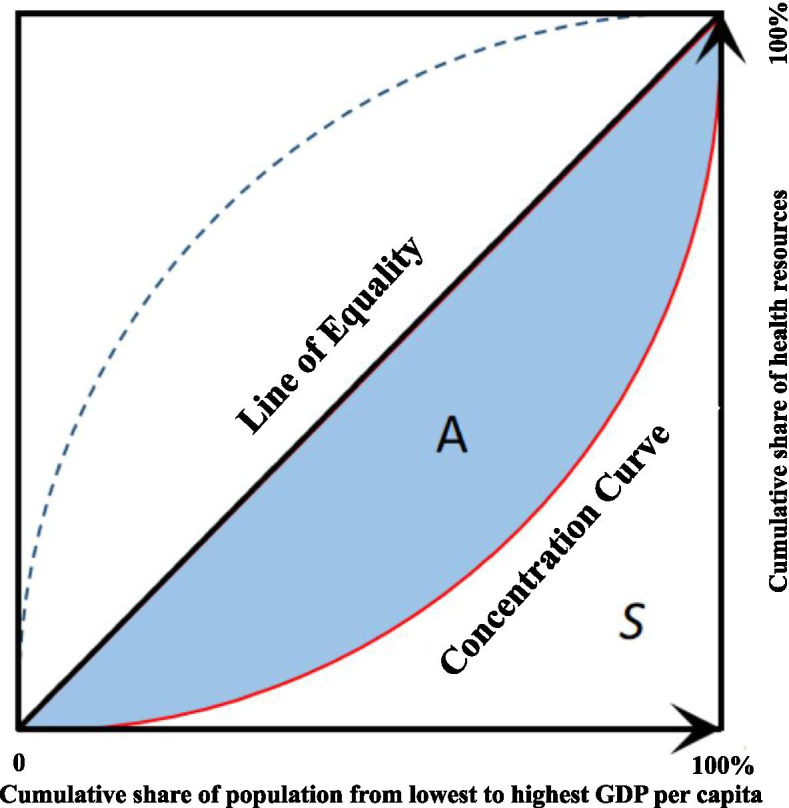
Graphical definition of CI

The x-axis is the cumulative share of the population, ordered by GDP per capita from lowest to highest, and the y-axis represents the cumulative share of health resources. The concentration curve presents the cumulative share of the health resources against the cumulative share of the population. *A* in Fig. 1 is the area between the line of equality (the 45° line) and the concentration curve. *S* is the area under the concentration curve. The CI is calculated as twice the area *A*. The following exact computational formula was used to calculate the CI.

$$\begin{aligned}S=&\frac{1}{2}\sum_{i=0}^{n-1}\left(Y_i+Y_{i+1}\right)\left(X_{i+1}-X_i\right)\\ CI=&2\ast\left(0.5-S\right)\end{aligned}$$

The CI is bound from -1 to +1. Where 0 means complete equity, and ±1 means complete inequity. A negative value indicates health resources are disproportionately concentrated among the poorer, whereas a positive value means that health resources are disproportionately concentrated within wealthier populations.

A Geo-Detector model is used to measure structured spatial heterogeneity of health resources and determine whether the health resources within strata are more similar than that between strata. Given the q-statistic calculated by the Geo-Detector model, we can say that 100q% information of health resources can be explained by structured heterogeneity based on the independent variable (the GDP per capita here). The q-statistic has a range of 0 to 1 and can be calculated as follows [36, 37]:

$$q=1-\frac{{\sum }_{h=1}^{L}{N}_{h}{\sigma }_{h}^{2}}{N{\sigma }^{2}}$$

Where *L* is the number of strata of GDP per capita, ${N}_{h}$ and *N* is the number of provinces in strata *h* and the number of all provinces, respectively, and ${\sigma }_{h}^{2}$ and ${\sigma }^{2}$ are the variance of resources in strata *h* and all data. ${\sum }_{h=1}^{L}{N}_{h}{\sigma }_{h}^{2}$ is within sum of square and $N{\sigma }^{2}$ is total sum of squares. Our research divided GDP per capita into four grades using lower quartile, median, and upper quartile (*L*=4).

However, the calculation of q-statistics needs to discretize the independent variables into the qualitative variable, which may cause the loss of information. Therefore, we used GWR to analyze the impact of economic factors on the distribution of health resources. In our study, Gaussian kernel was used, and the optimal bandwidth was selected using leave-one-out cross validation.

R studio software (version 4.1.1) was used to perform all analyses. *P* < 0.05 was considered statistically significant.

## Results

### Overview of health resources over the past ten years

Changes in the number of health resources in China from 2009 to 2018 are shown in Table 1. Generally, health resources, including healthcare institutions, beds, health personnel, and health expenditures have increased over the past ten years. Important indicators for the allocation of resources in the global study of health-service systems such as beds per 1,000 people and HCE per 1,000 people show a steady growth trend. Compared with 2009, numbers in 2018 increased by 83.86% and 51.11%, respectively. The health investment of the Chinese government is also growing. Over the past decade, GHE has grown steadily at an annual growth rate of 11.88%. Correspondingly, the GHE per capita in 2018 increased by 2.6 times compared with 2009. The structure of health expenditure has also improved. The proportion of OOPs in THE continued to decrease, i.e., from 37.46% to 2009 to 28.61% in 2018, resulting in the lowest level in nearly ten years. Meanwhile, the proportion of SHE in THE has increased from 35.08% to 2009 to 43.66% in 2018.

**Table 1:** Overall condition of health resources in China from 2009 to 2018

	Institutions		Beds			Health Personnel				Health Expenditure					
Year	Hospitals	PMHI	Hospitals	PMHI	Beds per 1,000 people	LD	RN	HCE	HCE per 1,000 people	GHE^a^(100 million yuan)	GHE per captial^a^	SHE^a^(100 million yuan)	OOPs^a^(100 million yuan)	OOPs(% of THE)	THE(% of GDP)
2009	20,291	882,153	3,120,773	1,099,662	3.16	1,905,436	1,854,818	7,781,448	5.83	5972.16	447.52	7631.57	8148.32	37.46%	5.03%
2010	20,918	901,709	3,387,437	1,192,242	3.42	1,972,840	2,048,071	8,207,502	6.12	6993.64	521.56	8779.86	8602.18	35.29%	4.84%
2011	21,979	918,003	3,705,118	1,233,721	3.67	2,020,154	2,244,020	8,207,502	6.09	8733.09	648.17	9847.25	9917.11	34.80%	4.98%
2012	23,170	912,620	4,161,486	1,324,270	4.05	2,138,836	2,496,599	9,115,705	6.73	9528.14	703.68	11334.69	10911.20	34.34%	5.20%
2013	24,709	915,368	4,578,601	1,349,908	4.36	2,285,794	2,783,121	9,790,483	7.20	10500.39	771.68	12533.17	11812.98	33.90%	5.32%
2014	25,860	917,335	4,961,161	1,381,197	4.64	2,374,917	3,004,144	10,234,213	7.48	11319.78	827.58	14378.39	12087.70	31.99%	5.48%
2015	27,587	920,077	5,330,580	1,413,842	4.91	2,508,408	3,241,469	10,693,881	7.78	13223.80	962.00	17497.11	12713.15	29.27%	5.95%
2016	29,140	926,518	5,689,000	1,441,900	5.16	2,651,398	3,507,166	11,172,945	8.08	14466.72	1046.26	19860.55	13871.65	28.78%	6.23%
2017	31,056	933,024	6,120,500	1,528,500	5.50	2,828,999	3,804,021	11,748,972	8.45	15509.99	1115.76	22703.99	15434.73	28.77%	6.36%
2018	33,009	943,639	6,519,800	1,583,500	5.81	3,010,376	4,098,630	12,290,325	8.81	16399.13	1175.24	25810.78	16915.89	28.61%	6.57%
Annual growth rates	5.56%	0.75%	8.53%	4.13%	6.99%	5.21%	9.21%	5.21%	4.69%	11.88%	11.32%	14.50%	8.45%	..	..

Abbreviations: *PMHI *Primary Medical and Health Institutions, *GHE *Government Health Expenditure, *SHE *Social Health Expenditure, *OOPs *Out-of-Pocket Payments, *THE *Total Health Expenditure, *LD *Licensed doctors, *RN *Registered Nurses, *HCE *Health Care Employees;^a^Figures adjusted for inflation and calculated in Chinese yuan in 2018 prices

### Spatiotemporal distribution of health resources

**Fig. 2:**
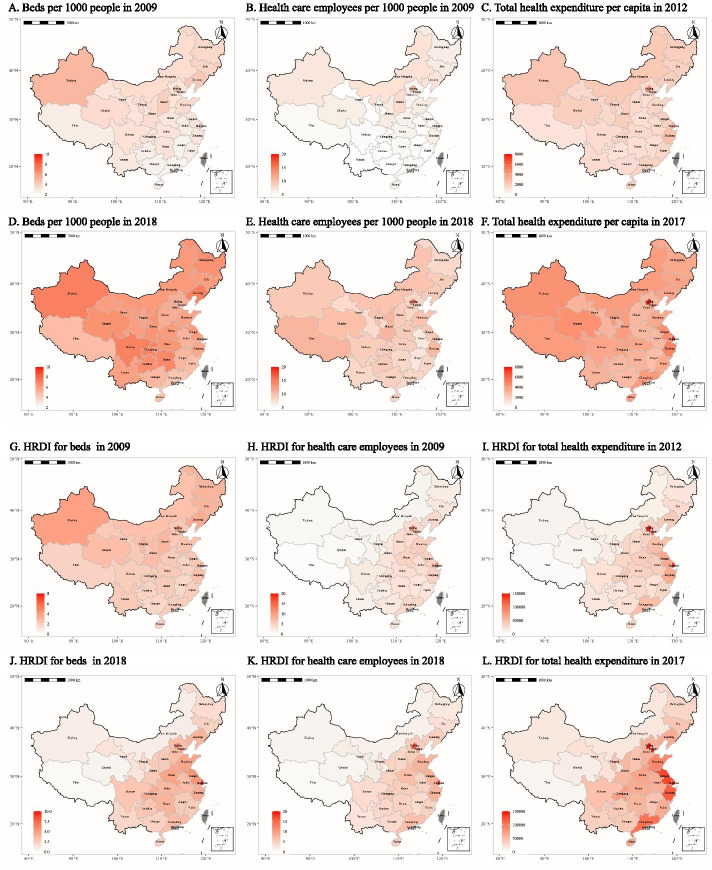
Comparison of health-resource distribution based on population density and HRDI

In general, the health resources (beds per 1,000 people, THE per capita, and HCE per 1,000 people) shown in Fig. 2 A-F indicated a remarkable increase, respectively. However, health-resource disparity existed among different regions in both years. Economically developed areas such as Beijing, Shanghai, Tianjin, Jiangsu, and Zhejiang were wealthier in health resources than other places. Some exceptions existed. Although Xinjiang was relatively backward in economic development (GDP per capita ranked 21st and 19th out of 31 provincial areas in 2009 and 2018, respectively), it had the highest number of beds per 1,000 people in China in 2009 and 2018 (Fig. 2-A and -D). The results of HRDI are shown in Fig. 2G-L. After adjusting for the geographical area, although disparities in the distribution of health resources remained, the distribution situation changed. Developed areas such as Beijing, Shanghai, Tianjin, Jiangsu, and Zhejiang still had higher values. However, in regions with large geographical areas such as Xinjiang and Tibet, their advantages in the distribution of health resources no longer exist. The results of each indicator are shown in Additional file 2 A-C.

Based on the calculation results of the global Moran’s I index, we can see a spatial disparity in the distribution of the health resources from 2009 to 2018 (Moran’s I >0 and P<0.05, Table 2). This finding was consistent with the conclusion of uneven distribution of HRDI shown in Fig. 2G-L. The results of local Moran’s I are shown in Table 3. Due to space constraints, Table 3 only shows the results of some provinces; please refer to Additional file 3 for detailed results. It can be seen that Beijing, Shanghai, and Tianjin had spatial correlation in most years, and the central and surrounding areas were both higher than the overall level (H, H). The local Moran’s I in Jiangxi, Jiangsu, Zhejiang, Xinjiang, and Tibet had statistical significance in some years. The first three provinces were (H, H) (like Beijing and Shanghai). Xinjiang and Tibet were (L, L), indicating that the indicators of the two provinces themselves and surrounding provinces were lower than the average.

**Table 2:** The global Moran’s I of HRDI for health resources in different years

HRDI	2009	2010	2011	2012	2013	2014	2015	2016	2017	2018
Beds	0.047^#^	0.0389^*^	0.054^#^	0.077^#^	0.079^#^	0.073^#^	0.081^#^	0.095^#^	0.097^#^	0.096^#^
Health personnel	0.055^#^	0.051^#^	0.070^#^	0.077^#^	0.080^#^	0.077^#^	0.083^#^	0.105^#^	0.105^#^	0.103^#^
Health expenditure	-	-	-	0.071^#^	0.073^#^	0.078^#^	0.079^#^	0.065^#^	0.057^#^	-

*: *P *< 0.05; #: *P *< 0.01

**Table 3:** The local Moran’s I of HRDI for health resources in different years

Area	Beds		Health personnel		Health expenditure	
	2009	2018	2009	2018	2012	2017
Beijing	0.923*(H,H)	1.637	1.245*(H,H)	3.529**(H,H)	1.477**(H,H)	2.361*(H,H)
Jiangxi	1.355	-0.076	1.375	0.063	1.810*(H,H)	0.108
Jiangsu	0.179	1.777*(H,H)	0.18	1.003	0.221	0.627
Shanghai	0.371	4.250**(H,H)	0.564	3.171*(H,H)	5.894**(H,H)	1.905*(H,H)
Tianjin	1.755*(H,H)	1.643	2.358*(H,H)	3.467*(H,H)	1.282**(H,H)	2.651*(H,H)
Tibet	0.731	0.235*(L,L)	0.775	0.18	0.47	0.081
Xinjiang	0.784	0.914*(L,L)	0.726	0.655*(L,L)	0.207	0.26
Zhejiang	0.022	1.821*(H,H)	0.046	1.907*(H,H)	0.094	1.11

*: *p *< 0.05; **: *p *< 0.001

### Equity in the allocation of health resources and the association between GDP per capita and health resources

**Fig. 3:**
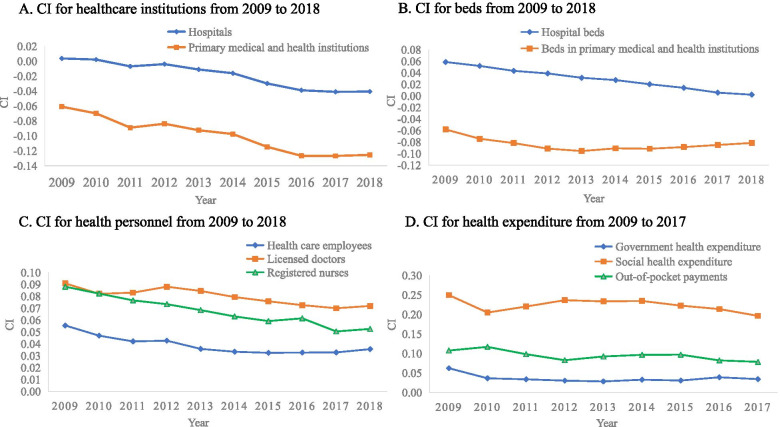
Trends of CI for ten indicators

The description of CI comprised four parts: healthcare institutions, beds, health personnel, and health expenditure. The trend of each indicator is shown in Fig. 3. The distribution of hospitals was relatively fair, with CI values close to 0(CImax= 0.0037, CImin= -0.0406). PMHI had always been primarily distributed in poorer areas, and the trend was an increasing one (from CI=-0.0646 in 2009 to CI=-0.0847 in 2018) (Fig. 3-A). Hospital beds tended to be distributed in wealthier areas, but this inequity decreased yearly (from CI=0.0587 in 2009 to CI=0.0021 in 2018). Beds in PMHI were primarily distributed in poorer areas. From 2009 to 2013, this kind of inequity expanded (from CI=-0.0581 in 2009 to CI=-0.0955 in 2013). Since 2013, this trend has eased (Fig. 3-B). Overall, the distribution of hospital beds and PMHI beds tended to be fair gradually, CI values tended to be close to 0. HCE was primarily distributed in the wealthier areas, but the overall trend of CI values was downward (from CI=0.0555 in 2009 to CI=0.0356 in 2018). LD (from CI=0.0909 in 2009 to CI=0.0719 in 2018) and RN (from CI=0.0881 in 2009 to CI=0.0526 in 2018) had the same trend, but their distribution was more unfair (Fig. 3-C). Health expenditure, including GHE, SHE, and OOPs were primarily concentrated in wealthier areas, with relatively stable positive CI values. The inequity degree of SHE was the highest (from CI=0.2496 in 2009 to CI=0.1964 in 2017) (Fig. 3-D). Meanwhile, GHE had the lowest level of CI, indicating a relatively fair distribution compared with the other two types of health expenditure.

After calculation, the q-statistics were around 0.29 to 0.51 with statistical significance expected for the beds in 2014 to 2016(the P values were 0.052, 0.062 and 0.062, respectively; Table 4). From the perspective of vertical years, the q-statistics of health resources increased, with beds increasing from 0.363 to 2009 to 0.401 in 2018, health personnel increasing from 0.412 to 2009 to 0.510 in 2018, and health expenditure increasing from 0.378 in 2012 to 0.425 in 2017. The increasing trend of q-statistics indicated that the matching degree between the distribution of health resources and GDP per capita was gradually improving. In a horizontal comparison of the three health resources indicators, the q-statistics of health personnel and health expenditure were higher than that of beds every year, indicating that GDP per capita had a more significant impact on the distribution of health personnel and health expenditure than beds. Table 5 shows the GWR results of health resources in 10 years. The table included the overall regression coefficient, minimum and maximum values of the GWR coefficient, and adjustment R^2^ of GWR. The results showed that all the GWRs were statistically significant, and the GWR coefficients in different areas in each GWR fluctuated little. The minimum and maximum of the coefficients were close to the overall regression coefficient. The adjusted R^2^ of GWR mainly concentrated from 0.5 to 0.7, indicating that economic factors closely impacted on health resources.

**Table 4:** The q-statistics of HRDI in different years

HRDI	2009	2010	2011	2012	2013	2014	2015	2016	2017	2018
Beds	0.363^*^	0.343^*^	0.340^*^	0.339^*^	0.322^*^	0.305	0.291	0.291	0.407^*^	0.401^*^
Health personnel	0.412^*^	0.389^*^	0.387^*^	0.387^*^	0.365^*^	0.337^*^	0.362^*^	0.366^*^	0.503^#^	0.510^#^
Health expenditure	-	-	-	0.378^*^	0.376^*^	0.371^*^	0.384^*^	0.378^*^	0.425^#^	-

*: *P *< 0.05; #: *P *< 0.01

**Table 5:** The results of GWR of health resources in 10 years

Year	Beds	Health personnel	Health expenditure
2009	0.08**	0.165**	-
	(0.062~0.085,0.669)	(0.143~0.173,0.727)	
2010	0.072**	0.146**	-
	(0.055~0.077,0.618)	(0.127~0.153,0.685)	
2011	0.062**	0.131**	-
	(0.063~0.073,0.54)	(0.133~0.154,0.629)	
2012	0.059**	0.127**	70829.639**
	(0.059~0.065,0.473)	(0.129~0.14,0.579)	(69154.848~79726.965,0.573)
2013	0.057**	0.121**	74987.434**
	(0.058~0.064,0.476)	(0.123~0.146,0.577)	(76728.511~87267.249,0.579)
2014	0.056**	0.12**	81311.573**
	(0.057~0.066,0.482)	(0.122~0.143,0.582)	(83350.619~93382.926,0.613)
2015	0.056**	0.122**	89729.788**
	(0.057~0.063,0.494)	(0.124~0.141,0.611)	(90274.719~100659.859,0.632)
2016	0.055**	0.119**	96768.578**
	(0.05~0.055,0.545)	(0.116~0.119,0.649)	(94117.045~97879.254,0.645)
2017	0.055**	0.12**	100961.734**
	(0.05~0.054,0.565)	(0.118~0.121,0.701)	(98550.737~102123.025,0.657)
2018	0.052**	0.119**	**-**
	(0.048~0.052,0.543)	(0.117~0.12,0.707)	

*: *p* < 0.05; **: *p* < 0.001

## Discussion

This was a longitudinal study that assessed the changes in health resources in China in the context of new healthcare reform for ten years. From 2009 to 2018, the total amount of health resources in China showcased a steady increase. Previous related studies focusing on the different periods have similar results [3, 40]. A review study about the ten years of healthcare reform in China has shown that the annual growth rate of both THE per capita and GHE per capita is higher than that of GDP per capita in the same timeframe [10]. This mismatch implies that the growth in health resources is due to economic growth and the increasing emphasis of Chinese governments on healthcare systems. In the early stages of the reform, the government significantly increased financial investment in the health sector. Liu et al. showed that from 2009 to 2013, health investment had grown considerably, indicating a twofold increase [3]. The level of health investment in China has considerably improved, but the proportion of THE in GDP remains lower than that in many countries [41]. Except for the financial investment, the Chinese government gradually turns its emphasis to healthcare-delivery reform [10]. Policies such as zero-markup drug policy, patient-referral policy, medical alliance policy, and reform of the medical-insurance payment system have been adopted to promote the reform. We found another important improvement was the positive change in the structure of health expenditure. The share of OOPs in THE has continued to decline, indicating that the problems of catastrophic health expenditures and impoverishment caused by limited health resources were alleviated. The possible reasons were as follows: First, China vigorously developed social medical insurance and increased its coverage. Since 2013, the coverage has remained above 95% [15]. Second, the government has gradually expanded the medical services covered by social medical insurance, reduced the coinsurance ratio, and increased the maximum reimbursements [42]. Third, the central government advocated the reform of medical-insurance payment methods [43]. New payment methods including global budgets, diagnosis-related groups, and case-based payments were applied to pilot experiments, which has been preliminarily proven to be effective in reducing OOPs [10]. In order to achieve the goal of reducing the share of OOPs in THE to 25% by 2030 [13], the Chinese government needs to continue to deepen the reform, including but not limited to the above aspects. It is worth noting that although the situation of health resources has improved significantly, there has also been an increase in the health needs of the people. For example, the number of RN per 1,000 people peaked at 2.94 in 2018, which is still far behind the target of 4.7 for Healthy China 2030 [13].

Maps were used to visually display the distribution of health resources in each area throughout the research period. Figure 2 A-F shows an obvious increase in health resources, but the disparities between different regions still existed in both years. This kind of difference was measured based on population density. Large, sparsely populated regions such as Tibet and Xinjiang had a remarkable advantage. However, this finding was not consistent with the fact that the National Health Commission sent medical technicians to Xinjiang and Tibet for counterpart aid almost every year. This distribution of health resources did not match the economic level, either. For example, the GDP per capita of Guangdong and Fujian in 2018 was 86,412 Yuan, 91,197 Yuan, respectively, higher than those of Qinghai (47,689) and Sichuan (48,883) [44]. However, the number of beds per 1,000 people and the number of HCE per 1,000 people were lower than those of Qinghai and Sichuan (in 2018, the number of beds per 1,000 people was 4.3 in Guangdong, 4.6 in Fujian, 6.4 in Qinghai, and 7.0 in Sichuan; the number of HCE per 1,000 people was 8.1 in Guangdong, 8.1 in Fujian, 9.5 in Qinghai, and 8.9 in Sichuan). To better integrate the influence of population density and geographical area, we adopted HRDI for further comparison. The HRDI for beds, health expenditure, and health personnel in Shanghai and Beijing remained the first and second, respectively, whereas Xinjiang almost remained the third from the bottom. This finding differed from the situation measured by population density, in which Xinjiang ranked at the top. Previous studies had similar results [3, 9, 16, 17, 19]. Figure 2G-L shows that the distribution of health resources presented a general trend of gradual enhancement from west to east. Health resources were primarily concentrated in the economically developed eastern regions. This kind of distribution had positive spatial autocorrelation with statistical significance every year using the global Moran’s I index. The local Moran’s I results also indicated that there was the obvious spatial aggregation of health resources in economically developed areas such as Beijing and Shanghai, whereas health resources were scarce in Xinjiang, Tibet, and the poor surrounding areas. This verified the necessity of health poverty alleviation projects proposed by “Healthy China 2030” to increase support for the development of medical and health institutions in poor central and western regions [13].

Overall, since the new healthcare reform, the uneven distribution of health resources still existed, but the degree of unfairness had gradually decreased. The CI for hospitals gradually approached 0, indicating that the distribution of hospitals in various provincial regions was gradually equal. CI for PMHI gradually increased in a negative direction, meaning that they were primarily distributed in economically underdeveloped areas. This distribution may be reasonable as this was consistent with their positioning. CI for beds (in hospital and PMHI) gradually approached 0, indicating that the distribution of beds in all provincial areas was gradually equitable. Moreover, compared with the other three categories of health-resource distribution, beds distribution showed better fairness. This finding was consistent with previous research [3]. The THE directly indicates the level of the country’s investment in the health field. However, the distribution of health expenditure was the most unequal among the four categories of health resources. SHE was primarily distributed in wealthier areas, and it was more unequal than the other two types of health expenditure. This finding was partly due to the fact that the contribution amount and reimbursement ratio of social medical insurance in each provincial area differed. The more affluent areas had greater health-financing intensity. Their commercial medical insurance and private health resources were more abundant, and social donations were more abundant. CI for OOPs was positive, indicating that OOPs for health services were higher in the relatively high-income groups. Liu et al. found that a higher family income corresponded with a higher health expenditure [45]. This finding was consistent with the findings of our study. Health personnel tended to be distributed to wealthier places. The same conclusion has been drawn in previous studies [9, 46]. Other countries such as Thailand also face the same problems [47]. The Chinese government has been committed to addressing the problem of unequal distribution of health personnel, and measures including giving the primary medical technicians preference to professional title assessment, salary reform, and education have been taken to improve the equity [48]. The results can be reflected in the changes in CI for health personnel that the level of unfairness is gradually decreasing.

The q-statistics gathered from the Geo-Detector model indicated a match between GDP per capita and health resources. The growing trend of q-statistics indicated an increased matching degree between GDP per capita and health resources. In 2017, more than 50% of information of health personnel and 40% information of beds and health expenditure can be explained by GDP per capita with statistical significance. This finding was consistent with a previous study declaring that the GDP per capita had spatially positive clustered impacts on bed distribution [34]. However, their matching degree was still relatively low, with a maximum of about 50%, suggesting that there were still other factors that may affect the distribution of health resources. This study found that health personnel and health expenditure were more affected by GDP per capita than beds. Consequently, the government should consider more economic factors when considering the balanced distribution of health personnel and health expenditure. The results of GWR indicated that the GDP per capita was positively associated with health-resources distribution, which was complementary to the Geo-Detector model results. The government should be more alert about the result as the economic factor could widen the gap in health inequality because of its clustered impacts on health resources.

This study also has some limitations. In CI calculation, health-expenditure data in a few provinces in 2009, 2010, and 2011 were not published, and the health expenditure of each province in 2018 was not yet available. Instead, we selected the data in 2012 and 2017 to calculate CI for qualitative judgment. Missing data may have different effects on the results. In addition, due to the lack of a price index in the health sector, we cannot compare the prices of health services by region, which may have a potentially uncertain impact on equity comparisons. For another, we discussed the health-resource distribution at the provincial level, and the situation inside the province has not been revealed. In addition, this paper only preliminarily explored the association of economic factors with the distribution of health resources, and other factors that may affect the allocation of health resources need to be further studied.

## Conclusions

Since implementing the new healthcare reform, China’s health resources have shown significant growth in the ten years. The distribution of health resources showed spatial aggregation. Overall, the distribution of health resources tended to be equitable gradually. The distribution of beds was more equitable than other health resources. PMHI and their beds were concentrated mostly in poor areas. Health expenditure and health personnel tended to be concentrated in wealthier areas. SHE had the highest inequity. GDP per capita was positively associated with the spatially clustered distribution of health resources. Economic factors should be considered as an important factor in the balanced allocation of health resources. This study can provide decision makers with an improved understanding of the current situation of health-resource allocation. Equity and accessibility should continue to be an important consideration in the allocation of health resources by governments. How to optimize the allocation of health resources requires further and more comprehensive research.

## Supplementary Information

**Additional file 1.**

**Additional file 2.**

**Additional file 3.**

## Data Availability

Please contact corresponding authors for data requests.

## Abbreviations

GDP: Gross Domestic Product
CI: Concentration Index
HRDI: Health Resource Density Index
PMHI: Primary medical and health institutions
GHE: Government Health Expenditure
SHE: Social Health Expenditure
OOPs: Out-of-Pocket Payments
THE: Total Health Expenditure
HCE: Health Care Employees
LD: Licensed doctors
RN: Registered Nurses
GWR: Geographic Weighted Regression
