# Supplementary material for: Ten years of China’s new healthcare reform: a longitudinal study on changes in health resources
Source: BMC Public Health. 2021 Dec 13;21:2272. doi: 10.1186/s12889-021-12248-9 (PMC8670033; doi:10.1186/s12889-021-12248-9)
Supplement: Supplementary file 1 — Additional file 1. [file 12889_2021_12248_MOESM1_ESM.docx]

**Hospitals:** include general hospitals, hospitals specialized in traditional Chinese medicine, hospitals of integrated traditional Chinese and western medicine, affiliated hospital of medical college, ethnic hospitals, specialized hospitals and nursing hospitals, excluding specialized disease prevention and treatment institutes, maternal and child health care hospitals and convalescent hospitals.

**Primary medical and health institutions:** include community health service centers, community health service stations, urban health centers, township health centers, village clinics, outpatient departments and clinics (health centers).

**Beds:** refer to the fixed actual beds (non authorized beds) at year-end, including regular beds, simple beds, monitoring beds, beds which are disinfected and repairing, beds deactivated due to expansion or overhaul, not including neonatal beds, predelivery bed, inventory bed, observation beds, temporary beds and family accompany beds.

**Government health expenditure:** refers to the expenditure of the governments at all levels on medical and health care services, medical subsidies, health administration and health insurance management, and undertakings of family planning etc.

**Social health expenditure:** refers to all inputs of society except the government in public health including the expenditures on social medical security, commercial health insurance, private expenditure on operation of medical and health care, social donation and contribution, and income from administrative fees etc.

**Out-of-pocket Payments:** refers to expenditure in cash on various health services by rural and urban residents, including self payments of residents within the system of multi-medical insurance.

**Health care employee:** refer to all employees engaged in the health care institutions, including medical technical personnel, village doctors and assistants, other technical personnel, managerial and service staff.

**Licensed doctors:** refer to the medical workers who have obtained the licenses of qualified doctors and are employed in medical and health care institutions, excluding the licensed doctors engaged in management job.

**Registered nurses:** refer to the medical workers who are certified as registered nurses and are actually engaged in nursing, excluding nurses engaged in management job.

**Total Population:**refers to the total number of people alive at a certain point of time within a given area.

**GDP Per capita:** refers to the ratio of gross domestic product in a year to the total population.
