# Supplementary material for: Ten years of China’s new healthcare reform: a longitudinal study on changes in health resources
Source: BMC Public Health. 2021 Dec 13;21:2272. doi: 10.1186/s12889-021-12248-9 (PMC8670033; doi:10.1186/s12889-021-12248-9)
Supplement: Supplementary file 2 — Additional file 2. [file 12889_2021_12248_MOESM2_ESM.zip › Appendix 2A. The distribution of beds.pdf]

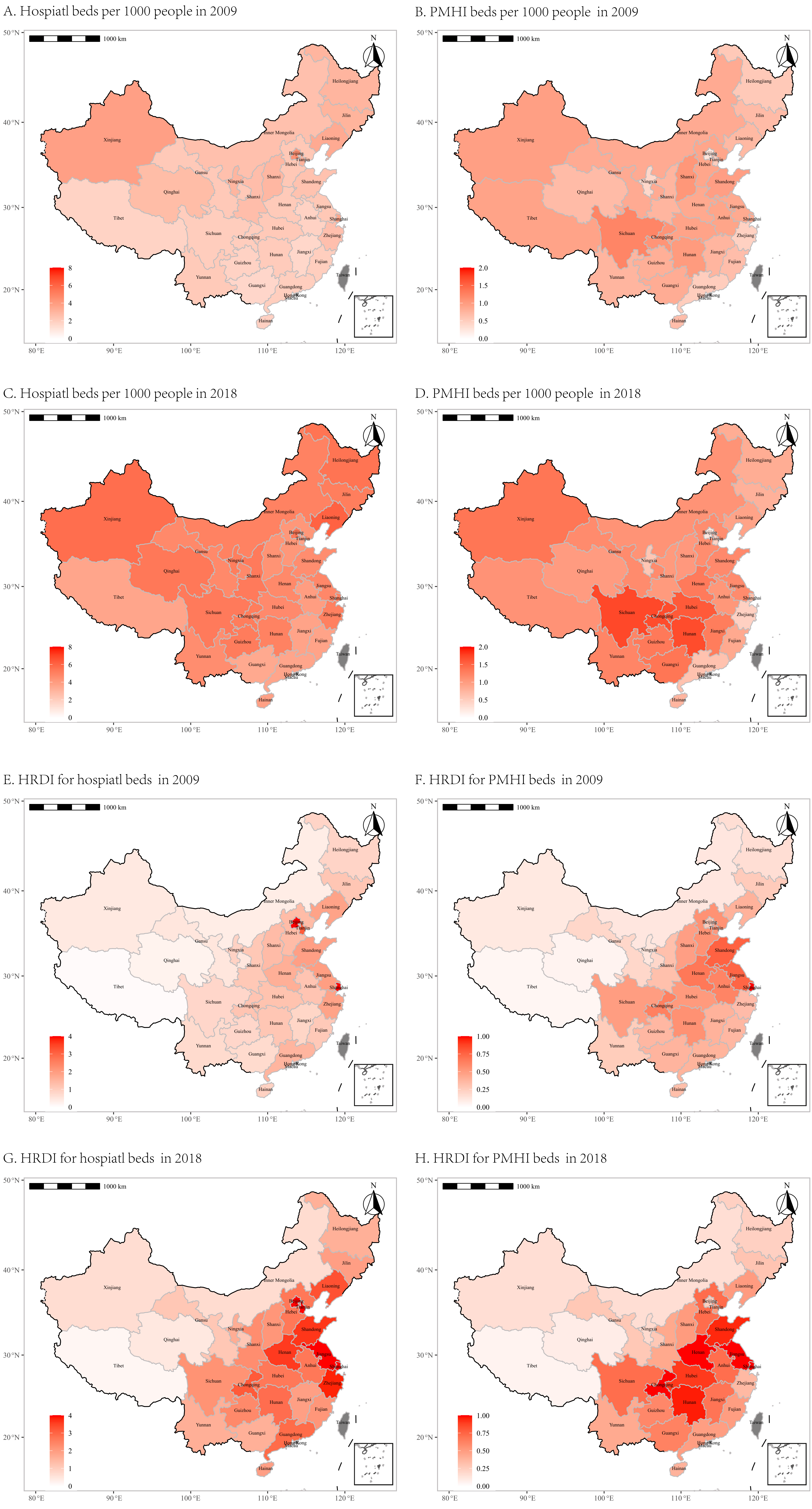
A. Hospiatl beds per 1000 people in 2009
B. PMHI beds per 1000 people in 2009
C. Hospiatl beds per 1000 people in 2018
D. PMHI beds per 1000 people in 2018
E. HRDI for hospiatl beds in 2009
F. HRDI for PMHI beds in 2009
G. HRDI for hospiatl beds in 2018
H. HRDI for PMHI beds in 2018
1000 km
N
50°N
40°N
30°N
20°N
80°E
90°E
100°E
110°E
120°E
Heilongjiang
Jilin
Liaoning
Inner Mongolia
Xinjiang
Beijing
Tianjin
Hebei
Gansu
Ningxia
Shanxi
Shandong
Qinghai
Shanxi
Henan
Jiangsu
Anhui
Shanghai
Tibet
Sichuan
Chongqing
Hubei
Zhejiang
Hunan
Jiangxi
Guizhou
Fujian
Taiwan
Yunnan
Guangxi
Guangdong
Hong Kong
Macau
Hainan
8
6
4
2
0
2.0
1.5
1.0
0.5
0.0
4
3
2
1
0
1.00
0.75
0.50
0.25
0.00
