# Supplementary material for: Ten years of China’s new healthcare reform: a longitudinal study on changes in health resources
Source: BMC Public Health. 2021 Dec 13;21:2272. doi: 10.1186/s12889-021-12248-9 (PMC8670033; doi:10.1186/s12889-021-12248-9)
Supplement: Supplementary file 2 — Additional file 2. [file 12889_2021_12248_MOESM2_ESM.zip › Appendix 2B. The distribution of health personnel.pdf]

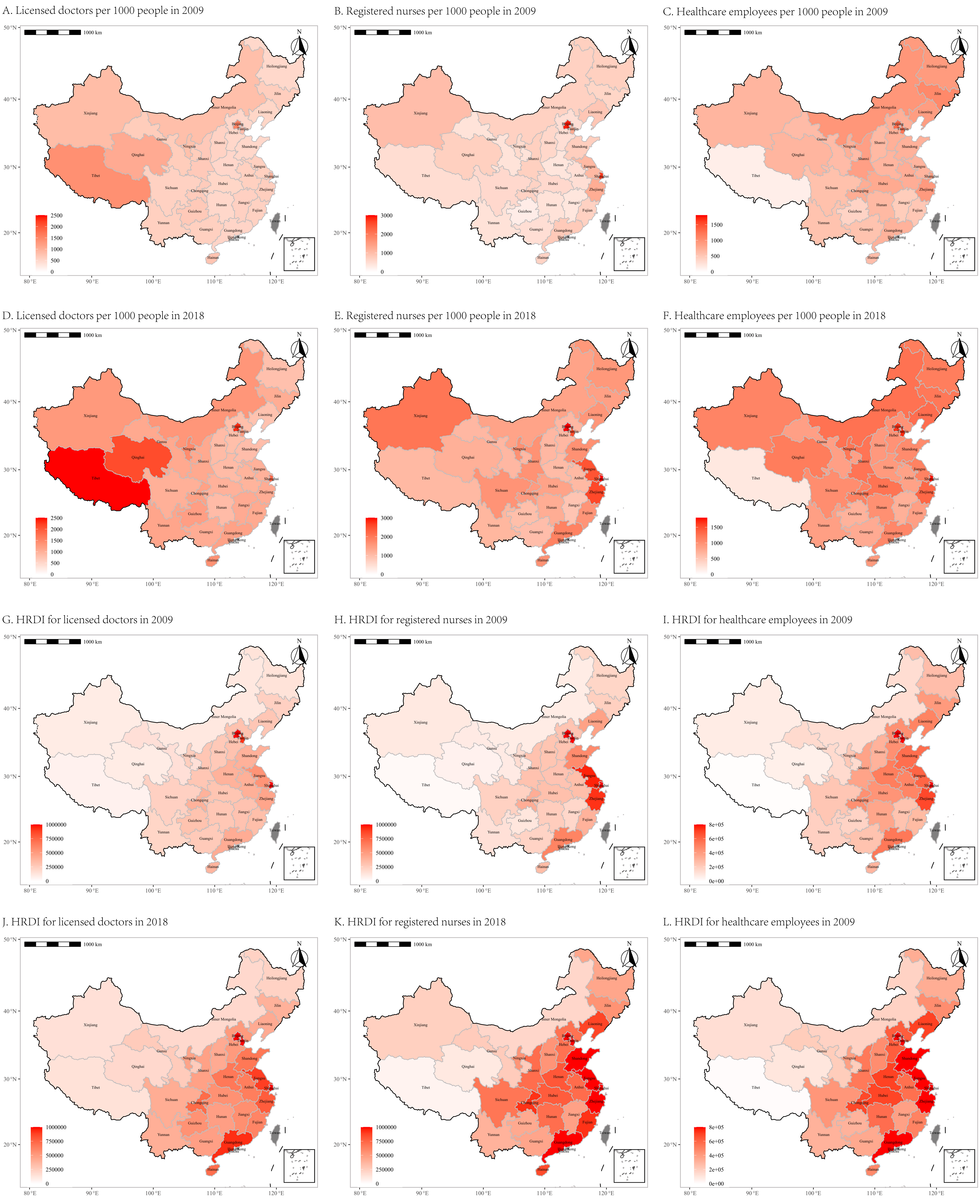
A. Licensed doctors per 1000 people in 2009
B. Registered nurses per 1000 people in 2009
C. Healthcare employees per 1000 people in 2009
D. Licensed doctors per 1000 people in 2018
E. Registered nurses per 1000 people in 2018
F. Healthcare employees per 1000 people in 2018
G. HRDI for licensed doctors in 2009
H. HRDI for registered nurses in 2009
I. HRDI for healthcare employees in 2009
J. HRDI for licensed doctors in 2018
K. HRDI for registered nurses in 2018
L. HRDI for healthcare employees in 2009
1000 km
N
2500 2000 1500 1000 500 0
3000 2000 1000 0
1500 1000 500 0
1000000 750000 500000 250000 0
8e+05 6e+05 4e+05 2e+05 0e+00
50°N 40°N 30°N 20°N
80°E 90°E 100°E 110°E 120°E
