# Supplementary material for: Ten years of China’s new healthcare reform: a longitudinal study on changes in health resources
Source: BMC Public Health. 2021 Dec 13;21:2272. doi: 10.1186/s12889-021-12248-9 (PMC8670033; doi:10.1186/s12889-021-12248-9)
Supplement: Supplementary file 2 — Additional file 2. [file 12889_2021_12248_MOESM2_ESM.zip › Appendix 2C. The disrtibution of health expenditure.pdf]

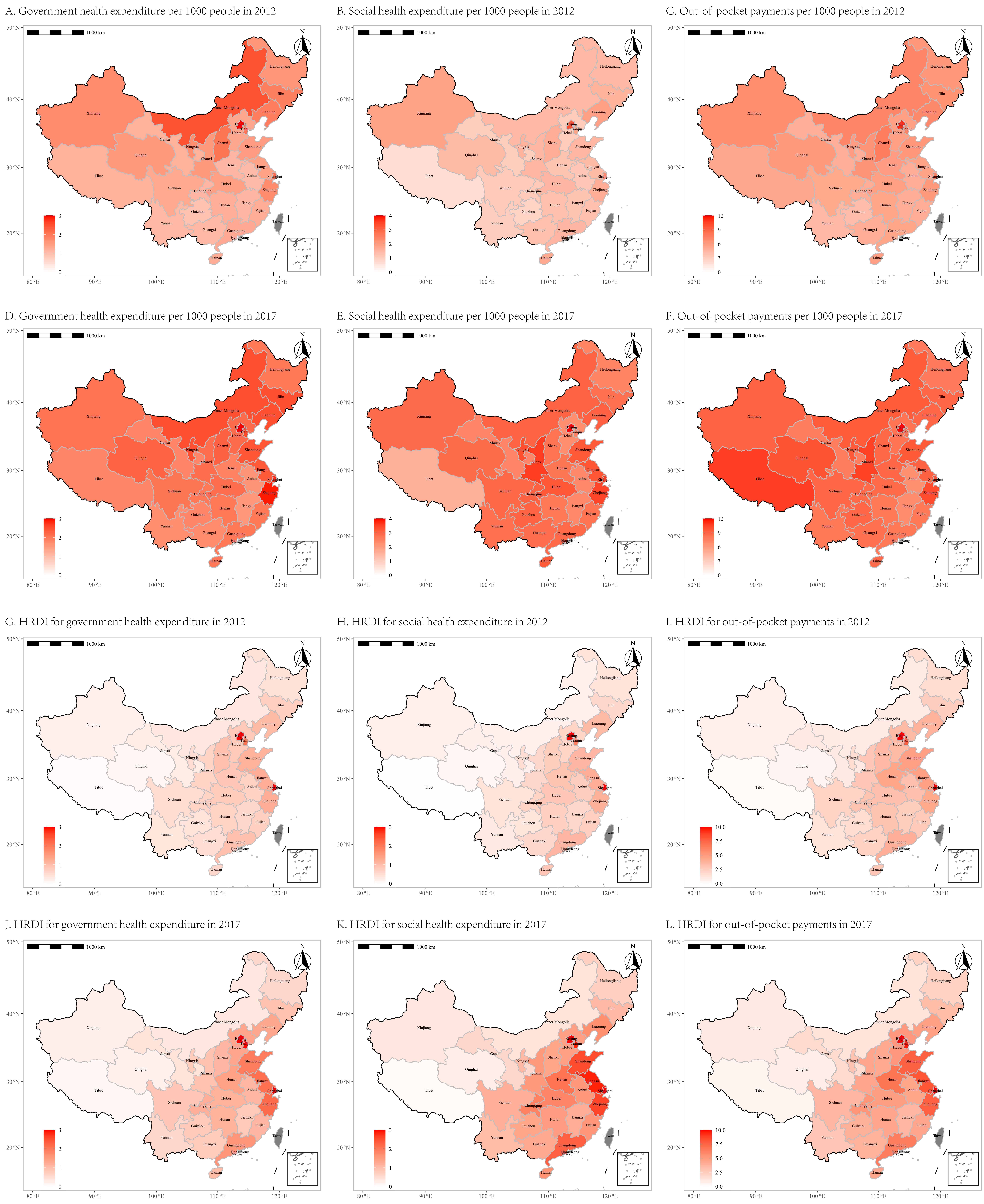
A. Government health expenditure per 1000 people in 2012
B. Social health expenditure per 1000 people in 2012
C. Out-of-pocket payments per 1000 people in 2012
D. Government health expenditure per 1000 people in 2017
E. Social health expenditure per 1000 people in 2017
F. Out-of-pocket payments per 1000 people in 2017
G. HRDI for government health expenditure in 2012
H. HRDI for social health expenditure in 2012
I. HRDI for out-of-pocket payments in 2012
J. HRDI for government health expenditure in 2017
K. HRDI for social health expenditure in 2017
L. HRDI for out-of-pocket payments in 2017
