# Supplementary material for: Ten years of China’s new healthcare reform: a longitudinal study on changes in health resources
Source: BMC Public Health. 2021 Dec 13;21:2272. doi: 10.1186/s12889-021-12248-9 (PMC8670033; doi:10.1186/s12889-021-12248-9)
Supplement: Supplementary file 3 — Additional file 3. [file 12889_2021_12248_MOESM3_ESM.docx]

Table 1. The local Moran’s I of HRDI for beds

| Province | 2009 | 2010 | 2011 | 2012 | 2013 | 2014 | 2015 | 2016 | 2017 | 2018 |
| --- | --- | --- | --- | --- | --- | --- | --- | --- | --- | --- |
| Anhui | 0.018 | 0.001 | -0.017 | -0.02 | -0.017 | -0.017 | -0.034 | 0.014 | 0.053 | 0.082 |
| Beijing | 0.923*  (H,H) | 0.899*  (H,H) | 4.479**  (H,H) | 1.228*  (H,H) | 1.705**  (H,H) | 1.750**  (H,H) | 1.672**  (H,H) | 2.018*  (H,H) | 1.821*  (H,H) | 1.637 |
| Fujian | -0.152 | -0.172 | -0.116 | -0.121 | -0.093 | -0.133 | -0.059 | -0.032 | -0.046 | -0.048 |
| Gansu | 0.827 | 0.59 | 0.647 | 0.875 | 0.86 | 0.447 | 0.991 | 1.424 | 1.425 | 1.435 |
| Guangdong | 0.004 | 0.017 | 0.029 | 0.05 | 0.044 | 0.038 | 0.028 | -0.017 | -0.022 | -0.02 |
| Guangxi | 0.397 | 0.42 | 0.469 | 0.465 | 0.458 | 0.495 | 0.102 | 0.13 | 0.17 | 0.164 |
| Guizhou | 0.536 | 0.506 | 0.499 | 0.462 | 0.323 | 0.231 | 0.203 | 0.038 | 0.017 | 0.014 |
| Hainan | 0.561 | 0.174 | 0.164 | 0.18 | 0.026 | 0.077 | 0.287 | 0.097 | 0.153 | 0.148 |
| Hebei | 0.002 | -0.004 | -0.013 | -0.015 | -0.007 | -0.009 | -0.004 | 0.001 | 0.027 | 0.036 |
| Henan | -0.068 | -0.33 | -0.474 | -0.594 | -0.457 | -0.461 | -0.752 | -0.01 | -0.003 | -0.002 |
| Heilongjiang | 0.036 | 0.302 | 0.345 | 0.383 | 0.377 | -0.163 | 0.146 | 0.075 | 0.096 | 0.075 |
| Hunan | 0.032 | 0.007 | 0.002 | -0.006 | -0.158 | -0.025 | -0.005 | -0.013 | 0 | -0.018 |
| Hubei | 0.091 | 0.057 | 0.008 | -0.008 | -0.015 | -0.078 | -0.141 | -0.027 | -0.017 | 0.006 |
| Jilin | -0.028 | -0.006 | -0.037 | -0.062 | -0.023 | -0.143 | -0.16 | 0.118 | 0.112 | 0.113 |
| Jiangxi | 1.355 | 1.409 | 2.960*  (H,H) | 3.565**  (H,H) | 4.204**  (H,H) | 4.362**  (H,H) | 4.351**  (H,H) | 1.611 | 1.728*  (H,H) | -0.076 |
| Jiangsu | 0.179 | 0.158 | 0.129 | 0.086 | 0.062 | 0.068 | 0.141 | -0.087 | -0.073 | 1.777*  (H,H) |
| Liaoning | -0.025 | 0.012 | 0.137 | 0.097 | 0.091 | 0.074 | 0.126 | -0.116 | -0.132 | -0.137 |
| Inner Mongolia | -0.092 | -0.11 | -0.131 | -0.075 | -0.33*  (L,L) | -0.345*  (L,L) | -0.311*  (L,L) | -0.011 | -0.026 | -0.03 |
| Ningxia | 0.319 | 0.327 | 0.337 | 0.348 | 0.523 | 0.215 | 0.431 | 0.419 | 0.426 | 0.445 |
| Qinghai | 0.681 | 0.022 | -0.028 | -0.155 | 0.44 | 0.155 | 0.644 | 1.481 | 1.461 | 1.491 |
| Shandong | -0.042 | -0.081 | 0.045 | -0.204 | -0.017 | 0.005 | 0.216 | 0.576 | 0.602 | 0.562 |
| Shanxi | -0.004 | 0 | 0.009 | 0.055 | 0.15 | 0.068 | 0.203 | 0.051 | 0.037 | 0.027 |
| Shaanxi | 0.14 | 0.004 | 0.008 | 0.204 | 0.037 | 0.125 | 0.21 | 0 | 0.005 | 0.005 |
| Shanghai | 0.371 | 0.294 | 2.527**  (H,L) | 5.770**  (H,H) | 6.316**  (H,H) | 6.549**  (H,H) | 6.512**  (H,H) | 3.812**  (H,H) | 4.143**  (H,H) | 4.250**  (H,H) |
| Sichuan | 0.169 | 0.151 | 0.122 | 0.082 | 0.066 | 0.077 | 0.129 | -0.052 | -0.049 | -0.05 |
| Tianjin | 1.755*  (H,H) | 1.746*  (H,H) | 0.271 | 1.075**  (H,H) | 0.677*  (H,L) | 0.648*  (H,L) | 0.625*  (H,L) | 2.162*  (H,H) | 1.887 | 1.643 |
| Tibet | 0.731 | 1.01 | 1.126 | 1.01 | 0.773 | 1.299 | 0.225 | 0.232*  (L,L) | 0.228*  (L,L) | 0.235*  (L,L) |
| Xinjiang | 0.784 | -0.071 | 0.283 | 0.135 | 0.548 | 0.614*  (L,L) | -0.214 | 0.878*  (L,L) | 0.885*  (L,L) | 0.914*  (L,L) |
| Yunnan | 0.432 | 0.673 | 0.682 | 0.474 | 0.481 | 0.546 | 1.056 | 0.118 | 0.065 | 0.055 |
| Zhejiang | 0.022 | 0.02 | 0.055 | 0.088 | 0.026 | 0.017 | 0.037 | 1.529 | 1.761*  (H,H) | 1.821*  (H,H) |
| Chongqing | 0.002 | -0.009 | -0.01 | -0.035 | -0.081 | -0.094 | -0.086 | -0.139 | -0.135 | -0.14 |
| *:*p*<0.05; **:*p*<0.001 | | | | | | | | | | |

Table 2. The local Moran’s I of HRDI for health personnel

| Province | 2009 | 2010 | 2011 | 2012 | 2013 | 2014 | 2015 | 2016 | 2017 | 2018 |
| --- | --- | --- | --- | --- | --- | --- | --- | --- | --- | --- |
| Anhui | 0.041 | 0.052 | 0.065 | 0.056 | 0.088 | 0.057 | 0.106 | -0.099 | -0.103 | -0.11 |
| Beijing | 1.245*  (H,H) | 1.328*  (H,H) | 5.742**  (H,H) | 1.478*  (H,L) | 2.248**  (H,H) | 2.329**  (H,H) | 2.391**  (H,H) | 3.581**  (H,H) | 3.661**  (H,H) | 3.529**  (H,H) |
| Fujian | -0.118 | -0.123 | -0.077 | -0.072 | -0.061 | -0.057 | -0.032 | -0.026 | -0.028 | -0.026 |
| Gansu | 1.042 | 0.642 | 0.642 | 1.052 | 1.041 | 0.69 | 1.005 | 1.17 | 1.131 | 1.133 |
| Guangdong | 0.05 | 0.044 | 0.035 | 0.087 | 0.111 | 0.113 | 0.044 | -0.05 | -0.052 | -0.057 |
| Guangxi | 0.365 | 0.349 | 0.34 | 0.319 | 0.296 | 0.289 | 0.115 | 0.069 | 0.073 | 0.069 |
| Guizhou | 0.766 | 0.747 | 0.726 | 0.686 | 0.612 | 0.535 | 0.345 | 0.166 | 0.148 | 0.133 |
| Hainan | 0.342 | 0.108 | 0.106 | 0.119 | 0.015 | 0.071 | 0.114 | 0.022 | 0.027 | 0.026 |
| Hebei | 0 | -0.006 | -0.002 | 0.013 | 0.011 | 0.013 | 0.008 | -0.031 | -0.02 | -0.009 |
| Henan | -0.087 | -0.324 | -0.46 | -0.442 | -0.3 | -0.359 | -0.575 | -0.007 | -0.01 | -0.011 |
| Heilongjiang | 0.047 | 0.307 | 0.409 | 0.428 | 0.424 | -0.15 | 0.276 | 0.204 | 0.232 | 0.233 |
| Hunan | 0.037 | 0.014 | 0.015 | 0.023 | 0.109 | 0.013 | 0.002 | 0.007 | 0.015 | 0.05 |
| Hubei | 0.178 | 0.141 | 0.062 | 0.078 | 0.044 | 0.101 | 0.076 | 0.036 | 0.042 | 0.024 |
| Jilin | 0.016 | 0.009 | 0.051 | 0.038 | 0.061 | 0.046 | -0.037 | 0.228 | 0.253 | 0.258 |
| Jiangxi | 1.375 | 1.503 | 2.366*  (H,H) | 2.515*  (H,H) | 2.743*  (H,H) | 3.024*  (H,H) | 3.040*  (H,H) | 0.917 | 0.934 | 0.063 |
| Jiangsu | 0.18 | 0.183 | 0.179 | 0.175 | 0.124 | 0.123 | 0.163 | 0.045 | 0.052 | 1.003 |
| Liaoning | -0.013 | 0.009 | 0.02 | 0.009 | -0.003 | -0.015 | -0.036 | 0.024 | 0.03 | 0.035 |
| Inner Mongolia | -0.106 | -0.13 | -0.208 | -0.134*  (L,L) | -0.23*  (L,L) | -0.244*  (L,L) | -0.222*  (L,L) | -0.151 | -0.141 | -0.14 |
| Ningxia | 0.384 | 0.399 | 0.321 | 0.313 | 0.53 | 0.183 | 0.28 | 0.343 | 0.32 | 0.317 |
| Qinghai | 0.709 | 0.047 | -0.088 | -0.134 | 0.35 | 0.079 | 0.655 | 1.196 | 1.155 | 1.155 |
| Shandong | -0.028 | -0.074 | -0.019 | -0.056 | 0.113 | 0.124 | 0.113 | 0.734 | 0.735 | 0.727 |
| Shanxi | -0.01 | -0.002 | 0.011 | 0.046 | 0.123 | 0.087 | 0.169 | 0.032 | 0.036 | 0.036 |
| Shaanxi | 0.131 | 0.028 | 0.021 | 0.155 | 0.046 | 0.089 | 0.119 | 0.004 | 0.004 | 0.004 |
| Shanghai | 0.564 | 0.482 | 2.298**  (H,H) | 5.867**  (H,H) | 6.193**  (H,H) | 6.591**  (H,H) | 6.413**  (H,H) | 2.969*  (H,H) | 3.011*  (H,H) | 3.171*  (H,H) |
| Sichuan | 0.195 | 0.204 | 0.201 | 0.18 | 0.168 | 0.074 | 0.267 | 0.019 | 0.017 | 0.01 |
| Tianjin | 2.358*  (H,H) | 2.481*  (H,H) | 0.615*  (H,L) | 1.177**  (H,H) | 0.635*  (H,L) | 0.634*  (H,L) | 0.633*  (H,L) | 3.555*  (H,H) | 3.631*  (H,H) | 3.467*  (H,H) |
| Tibet | 0.775 | 0.981 | 0.987 | 0.925 | 0.709 | 1.055 | 0.457 | 0.19 | 0.181 | 0.18 |
| Xinjiang | 0.726 | -0.086 | 0.276 | 0.15 | 0.589 | 0.524 | -0.121 | 0.649*  (L,L) | 0.648*  (L,L) | 0.655*  (L,L) |
| Yunnan | 0.544 | 0.968 | 0.982 | 0.569 | 0.55 | 0.575 | 1.142 | 0.302 | 0.272 | 0.257 |
| Zhejiang | 0.046 | 0.05 | 0.069 | 0.104 | 0.071 | 0.062 | 0.085 | 1.802*  (H,H) | 1.831*  (H,H) | 1.907*  (H,H) |
| Chongqing | 0.024 | 0.024 | 0.025 | 0.015 | 0.017 | 0.02 | 0.006 | -0.006 | -0.005 | -0.017 |
| *:*p*<0.05; **:*p*<0.001 | | | | | | | | | | |

Table 3. The local Moran’s I of HRDI for health expenditure

| Province | 2012 | 2013 | 2014 | 2015 | 2016 | 2017 | 2018 |
| --- | --- | --- | --- | --- | --- | --- | --- |
| Anhui | 0.073 | 0.127 | 0.099 | 0.176 | -0.071 | -0.07 | -0.07 |
| Beijing | 1.477**  (H,H) | 1.971**  (H,H) | 2.178**  (H,H) | 2.150**  (H,H) | 2.783*  (H,H) | 2.351*  (H,H) | 2.361*  (H,H) |
| Fujian | -0.013 | -0.014 | 0.001 | 0.015 | -0.021 | -0.02 | -0.02 |
| Gansu | 0.599 | 0.603 | 0.49 | 0.57 | 0.548 | 0.537 | 0.536 |
| Guangdong | -0.008 | -0.006 | 0.001 | -0.019 | -0.047 | -0.04 | -0.039 |
| Guangxi | 0.244 | 0.263 | 0.286 | 0.181 | 0.131 | 0.111 | 0.112 |
| Guizhou | 0.462 | 0.467 | 0.483 | 0.278 | 0.222 | 0.203 | 0.203 |
| Hainan | 0.16 | 0.171 | 0.175 | 0.127 | 0.068 | 0.053 | 0.054 |
| Hebei | 0.126 | 0.074 | 0.095 | 0.045 | -0.161 | -0.158 | -0.159 |
| Henan | 0.119 | 0.112 | 0.111 | 0.119 | 0.024 | 0.024 | 0.024 |
| Heilongjiang | 0.242 | 0.234 | 0.122 | 0.322 | 0.145 | 0.162 | 0.159 |
| Hunan | 0.05 | 0.2 | 0.048 | 0.034 | 0.079 | 0.076 | 0.106 |
| Hubei | 0.117 | 0.065 | 0.148 | 0.116 | 0.112 | 0.106 | 0.075 |
| Jilin | 0.068 | 0.063 | 0.086 | 0.02 | 0.152 | 0.167 | 0.164 |
| Jiangxi | 1.810*  (H,H) | 2.061*  (H,H) | 2.478*  (H,H) | 2.347*  (H,H) | 0.633 | 0.631 | 0.108 |
| Jiangsu | 0.221 | 0.146 | 0.22 | 0.231 | 0.114 | 0.108 | 0.627 |
| Liaoning | 0.007 | 0.003 | 0.008 | -0.008 | 0.04 | 0.048 | 0.047 |
| Inner Mongolia | -0.085 | -0.174 | -0.181 | -0.168 | 0.018 | 0.026 | 0.025 |
| Ningxia | 0.164 | 0.28 | 0.153 | 0.186 | 0.175 | 0.163 | 0.162 |
| Qinghai | 0.252 | 0.301 | 0.094 | 0.349 | 0.53 | 0.519 | 0.518 |
| Shandong | -0.015 | -0.005 | -0.001 | 0 | 0.099 | 0.054 | 0.054 |
| Shanxi | 0.107 | 0.267 | 0.203 | 0.215 | 0.139 | 0.14 | 0.14 |
| Shaanxi | 0.179 | 0.052 | 0.13 | 0.152 | 0.01 | 0.009 | 0.009 |
| Shanghai | 5.894**  (H,H) | 6.250**  (H,H) | 6.798**  (H,H) | 6.568**  (H,H) | 1.966*  (H,H) | 1.937*  (H,H) | 1.905*  (H,H) |
| Sichuan | 0.218 | 0.203 | 0.229 | 0.313 | 0.047 | 0.044 | 0.044 |
| Tianjin | 1.282**  (H,H) | 0.949*  (H,H) | 0.966*  (H,H) | 0.967*  (H,H) | 3.069*  (H,H) | 2.639*  (H,H) | 2.651*  (H,H) |
| Tibet | 0.47 | 0.429 | 0.555 | 0.407 | 0.083 | 0.081 | 0.081 |
| Xinjiang | 0.207 | 0.32 | 0.242 | 0.173 | 0.263 | 0.26 | 0.26 |
| Yunnan | 0.282 | 0.291 | 0.307 | 0.642 | 0.278 | 0.259 | 0.259 |
| Zhejiang | 0.094 | 0.045 | 0.049 | 0.051 | 1.158 | 1.136 | 1.11 |
| Chongqing | 0.025 | 0.018 | 0.034 | 0.019 | 0.063 | 0.061 | 0.062 |
| *:*p*<0.05; **:*p*<0.001 | | | | | | | |
